# “Cognition in marine mammals: the strength of flexibility in adapting to marine life”

**DOI:** 10.1007/s10071-022-01681-x

**Published:** 2022-09-24

**Authors:** Frederike D. Hanke, Kristy L. Biolsi, Heidi E. Harley

**Affiliations:** 1 Institute for Biosciences, Neuroethology, University of Rostock, Rostock, Germany; 2 St. Francis College, Brooklyn Heights, NY USA; 3 Center for the Study of Pinniped Ecology and Cognition (C-SPEC), Brooklyn Heights, USA; 4 New College of Florida, Sarasota, FL USA

## Abstract

In this theme issue, our multidisciplinary contributors highlight the cognitive adaptations of marine mammals. The cognitive processes of this group are highly informative regarding how animals cope with specifics of and changes in the environment, because, not only did modern marine mammals evolve from numerous, non-related terrestrial animals to adapt to an aquatic lifestyle, but some of these species regularly move between two worlds, land and sea. Here, we bring together scientists from different fields and take the reader on a journey that begins with the ways in which modern marine mammals (whales, dolphins, seals, sea lions and manatees) utilize their perceptual systems, next moves into studies of the constraints and power of individuals’ cognitive flexibility, and finally showcases how those systems are deployed in social and communicative contexts. Considering the cognitive processes of the different marine mammals in one issue from varying perspectives will help us understand the strength of cognitive flexibility in changing environments—in marine mammals and beyond.

Marine mammals, obligate air breathers that spend much or all of their time in the ocean, have been valuable experimental subjects of cognitive research for many decades with investigations of species from all major groups (i.e., cetaceans, pinnipeds, sirenians, mustelids, and ursids). Particularly, research with bottlenose dolphins (*Tursiops truncatus*) in human care has revealed astonishing cognitive capabilities, sometimes even rarely observed in other non-human animals (for an overview, see Herman 2010; Harley 2013). Nevertheless, marine mammal cognitive research has failed to strategically document how adaptation to the underwater realm has shaped cognition and how the marine mammals’ versatility in different environments has affected the flexibility of their cognitive processes.

Cognitive flexibility is critical to these animals that constantly experience changes in external parameters. These environmental changes occur naturally for example during dives, during migrations, or during transitions between air and water, but accelerate as climate continues to change and anthropogenic activities impact marine mammal habitats. It is the power of cognition, or specifically of cognitive flexibility, that allows the marine mammals to alter their behavior quickly to adapt in real time. Continued study of the sensory, perceptual, and cognitive systems of these non-human animals will thus shed more light on their adaptations to a marine life, also providing insights relevant to other animals as they too need to adapt. In this special issue, we have compiled articles describing cognitive flexibility/processes of different marine mammal species from varying perspectives. Our contributors approach the general theme through several major subthemes (perception and sensory integration, lab and field synergy, classic studies of cognitive abilities, neural substrates, and social interactions) in which they frame new developments in the field, review past research, or highlight ideas as well as novel experimental approaches for future research.

The special issue begins with perception, a critical starting point since the environments within which animals live is that which they perceive. Perception moreover forms the basis for all decision making. For example, one challenge is that noise may interfere with signal detection, discrimination or localization, a process called signal masking, which may thus significantly influence decision making and real-time behaviors. Branstetter and Sills (2022) review the extensive literature on the mechanisms of auditory masking in marine mammals critical for assessing the impact of external noise, natural as well as anthropogenic, on hearing in general and hearing-mediated behavior in particular. While auditory masking influences the perception of sound in the environment, species that are capable of echolocation have an additional perceptual analysis that can be utilized for processing sensory stimuli and making decisions in a fast-changing marine environment. Echolocating marine mammals such as the bottlenose dolphin seem to be able to actively shape how they perceive their environment by modulating their echolocation signals. Harley et al. (2022) document the dolphins’ differential investment in echoic analysis of auditory scenes depending on familiarity of the scenes and the animal’s expectations, underscoring once more (compare with for example Kloepper et al. 2014; Nachtigall and Supin 2008; Nachtigall et al. 2018) that echolocation is an active process. Whereas odontocetes produce echolocation signals, mysticetes lack this ability. However, mysticetes such as the humpback whale (*Megaptera novaeangliae*) produce remarkable vocalizations heard over many kilometers (Clapham 2018). Numerous researchers have speculated on how much vocal control humpback whales have over their versatile songs, but Mercado III et al. (2022) bring new evidence to bear suggesting that singing humpback whales indeed have volitional control over their songs and vary sequences over phrases and songs rather than merely (re)producing stereotyped songs.

While most research on perception deals with sensory modalities in isolation, marine mammals often rely on input from numerous modalities at once. Hence, it is important to assess how sensory systems work together and how sensory information is integrated (one of the main questions of sensory biology as formulated by Johnsen 2017) to form a multimodal representation of the environment. In this issue, Bruck and Pack (2022) highlight the value and importance of cross-modal studies to gain an integrated view of how marine mammals negotiate their world. The power of such multimodal approaches is exemplified in Charrier et al.’s (2022) work focusing on understanding complex behaviors such as mother–pup recognition in Australia sea lions *(Neophoca cinerea)*.

Charrier et al.’s work also illustrates that studies with animals under human care and field work need to go hand in hand to advance the field of marine mammal cognition. Studies with animals under human care can reveal mechanisms of perception (see for example in this issue Branstetter and Sills 2022) or describe basic cognitive abilities under controlled conditions (see for example in this special issue Loth et al. 2022; Manitzas-Hill et al. 2022). However, field work provides the required ecological context needed for a more complete and well-rounded understanding of complex behavior. Biolsi and Woo (2022), Bauer and Reep (2022), and Henaut et al. (2022) all stress the importance of such a “combined” approach, suggesting experiments with animals under human care and field work that move forward together right from the start (Biolsi and Woo 2022) or take anecdotal observations of behaviors of wild animals into consideration (Bauer and Reep 2022). They highlight that the study of cognitive processes is best served through as many perspectives as possible.

Our special issue then moves to classic cognitive experiments conducted with animals under human care. In a study specifically focused on flexible thinking, Manitzas-Hill et al. (2022) were able to teach killer whales (*Orcinus orca*) an “innovate cue” to which the whales either presented entirely novel behaviors or displayed a behavior not shown in the session before. Behavioral flexibility was also approached in two reversal learning experiments including harbor seals (*Phoca vitulina*) (Erdsack et al. 2022; Niesterok et al. 2022). These two studies draw attention to the power but also the limitations of behavioral flexibility in this species: whereas flexibility in the visual domain only occurs in single individuals, all tested harbor seals responded flexibly to spatial information. The latter finding mirrors large spatial requirements imposed on seals in their natural habitat when navigating in the open ocean or returning to foraging areas or haul-out sites. In contrast, vision might be occasionally impaired due to low ambient luminance or turbidity (but see new idea raised within Gläser et al. 2014), and correspondingly, visual cognitive abilities might not be as developed as spatial cognitive abilities. While previous cognitive studies including harbor seals, albeit successful, have often dealt with visual cognition (see for example Mauck and Dehnhardt 2005; Scholtyssek et al. 2013), we might gain a better understanding of cognitive abilities of harbor seals when addressing spatial cognition or at least when also considering spatial aspects (compare with Mauck and Dehnhardt 2007; Renouf and Gaborko 1988).

In another well-controlled experiment with animals under human care, Loth et al. (2022) revisited mirror-experiments as previous experiments were methodologically inconclusive (Morrison and Reiss 2018; Reiss and Marino 2001). According to the new study (Loth et al. 2022), bottlenose dolphins can use mirrors for self-inspection; nevertheless it is still hard to assess whether mirror-experiments can be interpreted regarding self-awareness (see Delfour 2006; Harley 2013). However, the authors’ observation that dolphins’ familiarity with natural reflecting surfaces such as the water’s surface (in line with Dibble et al. 2017)—one of their experimental animals seemed to have used the water surface instead of the artificial mirror to control its eye region during marking sessions—illustrates that specifics of the underwater environment, such as the presence of reflecting surfaces, may foster varying utilization of perceptual skills and different cognitive mechanisms related to the processing of stimulus representation and problem solving.

The specific conditions encountered underwater might have also affected neural adaptations. One of the most prominent differences between aerial and underwater habitats is the three-dimensionality of the underwater environment which allows for a high degree of freedom of movements, perhaps even more so than flying animals experience given how many marine mammals periodically swim upside down and rotate quickly around all body axes. Cook and Berns (2022) focus on the California sea lion’s (*Zalophus californianus*) brain and the size and connectivity of its caudate nucleus as putative neural substrate for three dimensional sensorimotor transformations. The sea lion’s caudate nucleus is large relative to brain volume, whereas the putamen is surprisingly small, and shows strong connections to other brain regions, unusually so when compared to other species. This study advances our understanding of the pinniped brain which has received relatively little scientific attention heretofore and raises a number of aspects such as the function of the caudate nucleus in respect to the three-dimensionality of the environment, to hunting behavior and even to cognitive flexibility, also discussed in three manuscripts in this special issue (Erdsack et al. 2022; Manitzas-Hill et al. 2022; Niesterok et al. 2022), to be researched in the future.

Our final section takes perceptual and cognitive processes into the social domain. Acquiring insight into mechanisms of conspecific interactions is critical to understanding marine mammals as many species are social, and all species interact for breeding purposes. In this special issue, entries on communication and social cognition move from a demonstration of conspecific gaze following in bottlenose dolphins (Johnson et al. 2022) to the nature of bonds between male dolphins (Fellner et al. 2022).

Overall, this issue brings together complementary aspects of work with marine mammals under human care and in the field, providing insight into the neural, perceptual, and cognitive structures of marine mammals as we understand them today. The contributions highlight that adaptation to the underwater realm has shaped cognition and that marine mammals possess cognitive flexibility on numerous levels—two avenues of marine mammal cognitive research worth pursuing further in future studies. From the contributions, we additionally learn that we should look for and expect species-specific cognitive adaptations and should document individual approaches to, or performances in, cognitive tasks (as reported in Erdsack et al. 2022; Loth et al. 2022; Manitzas-Hill et al. 2022). Observations of individuals/individuality can be inspiration for future experiments as evolution continues to act on individuals.

This special issue also helps to assess and stress the role of cognitive processes with respect to environmental changes. Numerous authors mention the need to include cognition in conservation plans or to consider cognitive abilities when assessing the impact of anthropogenic activities in the ocean, the habitat of the marine mammals. Gulland et al. (2022) note aspects such as “incomplete characterization of marine mammal biology and ecology,” (p.1), and “uncertainty in how individuals and populations would respond when confronted with profound changes in environmental conditions,” (p.1) that complicate predictions on how marine mammals will respond to climate change. The contributions of this special issue highlight the usefulness of understanding cognition and cognitive flexibility as central to real-time adaptation during this period of accelerating environmental changes. Thus, the more we know about marine mammal cognition—and there is still room for future research building on the inspiring studies reported in this special issue among others, the better we will be able to make predictions about our shared future fate in a changing world.

In conclusion, this special issue on marine mammal cognition presents variety within a shared focus and functions as a comprehensive source of literature as well as inspiration for experts both within and outside the field. We hope that your reading will offer many enjoyable moments of discovery.
